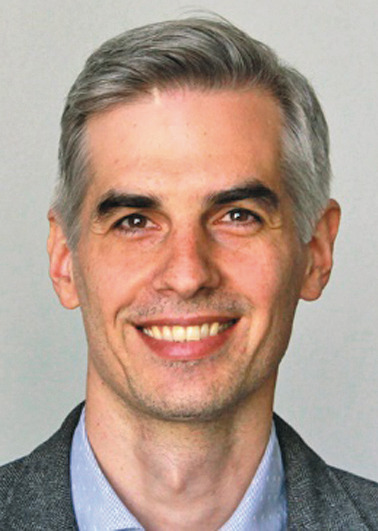# Exploring the Potential of Metal‐Based Candidate Drugs as Modulators of the Cytoskeleton

**DOI:** 10.1002/cbic.202300178

**Published:** 2023-08-01

**Authors:** Yasmin Borutzki, Lukas Skos, Christopher Gerner, Samuel M. Meier‐Menches

**Affiliations:** ^1^ Institute of Inorganic Chemistry Faculty of Chemistry University of Vienna 1090 Vienna Austria; ^2^ Department of Analytical Chemistry Faculty of Chemistry University of Vienna 1090 Vienna Austria; ^3^ Doctoral School of Chemistry University of Vienna 1090 Vienna Austria; ^4^ Joint Metabolome Facility University of Vienna and Medical University Vienna 1090 Vienna Austria

**Keywords:** actin, cytoskeleton, metals in medicine, plectin, tubulin, vimentin

## Abstract

During recent years, accumulating evidence suggested that metal‐based candidate drugs are promising modulators of cytoskeletal and cytoskeleton‐associated proteins. This was substantiated by the identification and validation of actin, vimentin and plectin as targets of distinct ruthenium(II)‐ and platinum(II)‐based modulators. Despite this, structural information about molecular interaction is scarcely available. Here, we compile the scattered reports about metal‐based candidate molecules that influence the cytoskeleton, its associated proteins and explore their potential to interfere in cancer‐related processes, including proliferation, invasion and the epithelial‐to‐mesenchymal transition. Advances in this field depend crucially on determining binding sites and on gaining comprehensive insight into molecular drug‐target interactions. These are key steps towards establishing yet elusive structure‐activity relationships.

## Graphical abstract

Metal‐based candidate drugs are promising modulators of cytoskeletal and cytoskeleton‐associated proteins. We summarise metal‐based candidate molecules that influence the cytoskeleton, its associated proteins and discuss their potential to interfere in cancer‐related processes.

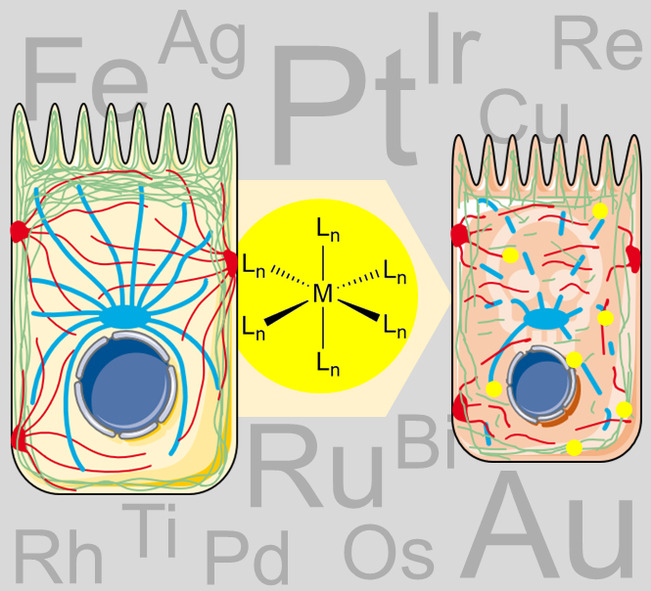

## 1 Introduction

Metal‐based drugs are a promising compound class for anticancer therapy^[1]^ and beyond.^[2]^ Due to their three‐dimensional coordination sphere, they are structurally unique compared to other small molecule drug classes and occupy a distinct area in the chemical space.^[3]^ They are often designed as prodrugs^[4]^ that activate upon reduction and/or ligand exchange. The activated species possess interesting chemo‐reactivities that can be best categorised as covalent inhibitors, due to their ability to coordinate to biological nucleophiles. However, they feature reversible properties depending on the off‐rate of ligand exchange between the metal and the target nucleophile.^[5]^

Metal‐based drugs are also a versatile class of therapeutically active compounds. The platinum(II) family members, cisplatin, carboplatin and oxaliplatin, are among the most famous metal‐based anticancer agents in clinical use.^[6]^ For the former two, platination of DNA and subsequent induction of apoptosis is key to their clinical efficacy against a range of tumours, including testicular, head and neck and ovarian cancer, amongst others. In contrast, oxaliplatin targets ribosome biogenesis^[7]^ and is effective in the therapy of cisplatin‐resistant tumours, *e. g*. in the adjuvant chemotherapy of stage III colon cancer.^[8]^ The metalloid arsenic trioxide relieves the differentiation blockade in acute promyelocytic leukaemia (PML) and achieves high cure rates.^[9]^ It binds to cysteine thiols on the PML‐moiety of the oncogenic fusion protein PML‐RARα, which leads to its degradation.^[10]^ A few examples of metal‐based drugs are in clinical trials, for instance, BOLD‐100 and ferroquine. The ruthenium(III) complex BOLD‐100 (formerly known as NKP‐1339, IT‐139) is investigated in a clinical phase Ib/IIa trial against gastrointestinal and pancreatic cancers in combination with FOLFOX.^[11]^ It shows a remarkable safety profile^[12]^ and features promising clinical outcomes (*NCT04421820*). Ferroquine is clinically evaluated against uncomplicated cases of malaria in combination with artefenomel with a phase IIb report available (*NCT02497612*).^[13]^ This ferrocene‐derivative of chloroquine makes use of the redox activity of ferrocene to impart orthogonal damage to the *plasmodium falciparum* parasite.^[14]^

Historically, metal‐based drug discovery is an area of phenotypic drug discovery for which identifying modes of action (MoA) has represented a serious bottleneck.^[15]^ There is a general consensus in the field that the metal‐centre and the ligand scaffold both dictate the biological effects of metal‐based candidate molecules.^[16]^ Yet, metal‐specific predictors for disease intervention remain to be elucidated. Mass spectrometry‐based techniques were recently developed that may facilitate discovery efforts by enabling MoA deconvolution and target identification in the cellular environment.[^5^, ^17^] Such approaches were successfully applied to a number of different metals and ligand scaffolds.^[18]^ These examples highlighted that even structurally simple compounds have the potential to specifically engage with novel targets and this property is not confined to particular metal centres. The biological effect of metal‐based candidate drugs is often accompanied by morphological changes and recently, a number of cytoskeletal and cytoskeleton‐associated proteins were validated as their targets.[^18b^, ^19^] This suggests that this compound class may have broad potential to modulate cytoskeletal and cytoskeleton‐associated proteins.

**Figure 1:**
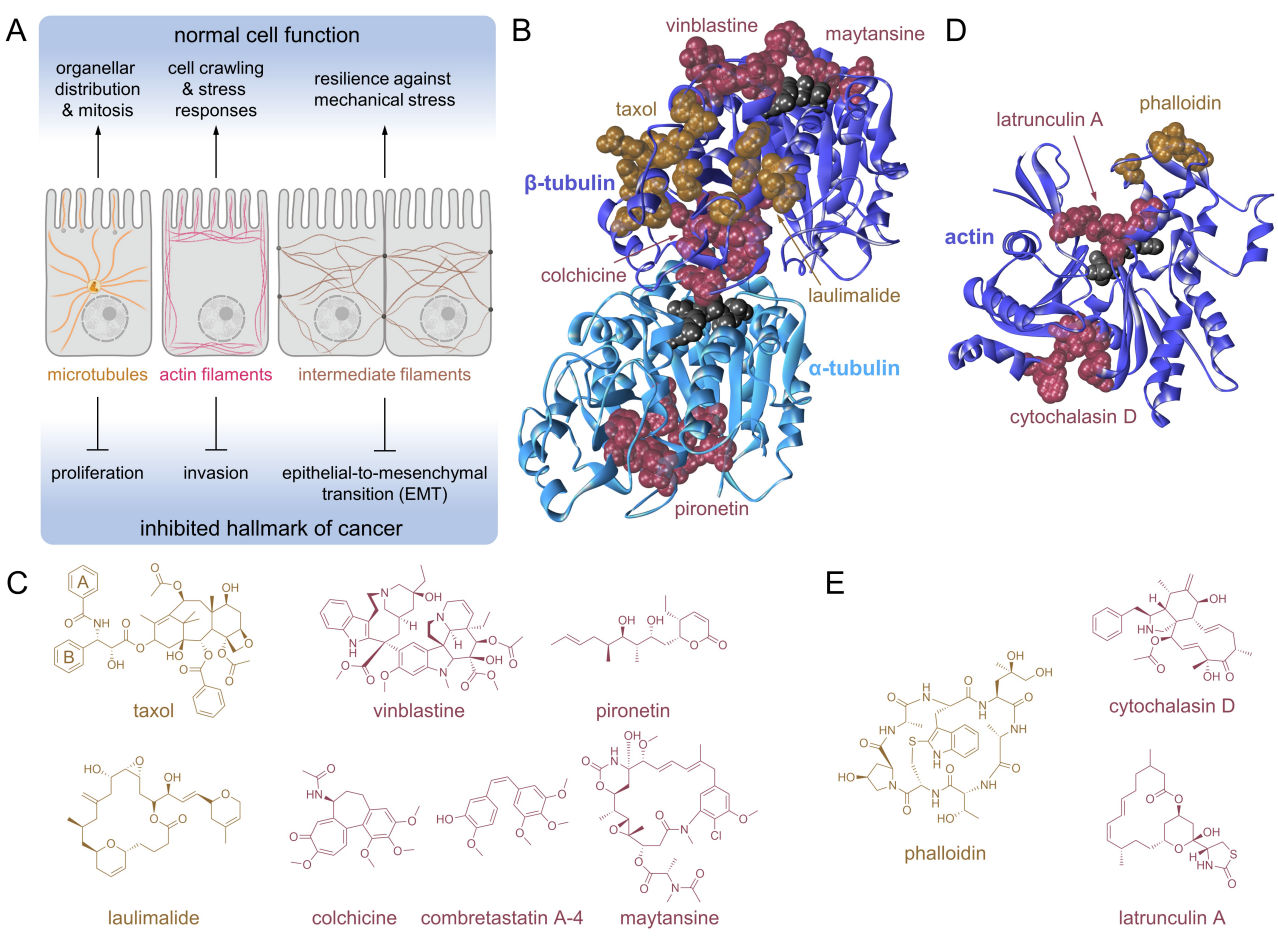
(**A**) Schematics of the distribution of the three cytoskeletal filaments are shown exemplarily in epithelial cells: microtubules, actin filaments and intermediate filaments (IFs) with connecting desmosomes as black circles. Microtubules organise organellar distribution and orchestrate mitosis. Actin filaments are involved in cell crawling, cell division and stress responses. IFs stabilise cells against mechanical stress. These cytoskeletal filaments can be pharmacologically targeted to disrupt cancer proliferation, invasion or the epithelial‐to‐mesenchymal transition (EMT). (**B**) Visualisation of the six established binding sites on α/β‐tubulin as space‐filling models of the involved amino acids. The taxol and laulimalide binding sites (gold) stabilise microtubules, while the vinblastine, maytansine, pironetin, colchicine (and its mimic CA‐4) binding sites (magenta) destabilise microtubules. GTPs are shown in grey. The structure was adapted from *PDB 1JFF*. (**C**) The chemical structures of the tubulin‐binding agents separated in microtubule stabilising (gold) and destabilising (magenta) compounds. (**D**) Visualisation of the three established binding sites on actin as space‐filling models of the involved amino acids. The phalloidin binding site (gold) stabilises actin filaments, while the latrunculin and cytochalasin binding sites (magenta) destabilise actin filaments. GTP is shown in grey. The structure was adapted from *PDB 1ESV*. (**E**) The chemical structures of the actin‐binding compounds separated into actin stabilising (gold) and destabilising (magenta) agents.

The cytoskeleton is a recognised drug target for anticancer therapy.^[20]^ It consists of three dynamic filamentous networks, including microtubules, actin filaments and intermediate filaments (IFs, Figure 1), as well as numerous cytoskeletal‐binding proteins.^[21]^ Microtubules organise organellar distribution, cell migration and cell cycle progression, including chromosome segregation during mitosis. These polarised filaments grow dynamically from a nucleating centre.^[20b]^ The actin filaments consist of single actin polymer strands that are responsible for cell crawling, cytoplasmic cleavage of dividing cells or stress responses by forming stress fibres.^[22]^ IFs form bundles and mesh‐like structures that stabilise cells against mechanical stress.^[20e]^ In epithelial cells, those are indirectly connected to the filaments of the neighbouring cells *via* desmosomes.^[23]^ IFs are implicated in the epithelial‐to‐mesenchymal transition (EMT), which is a relevant process in cancer progression.^[24]^ Some cytoskeletal proteins, including cytoskeleton‐associated proteins, are considered *hard‐to‐drug* targets^[25]^ because they may lack a clearly defined binding pocket.

Small molecules are inherently linked to the discovery of the cytoskeleton.^[26]^ It is noteworthy that tubulin was discovered as a consequence of searching for the target of the tropolone derivative colchicine,^[27]^ which was found to destabilise microtubules. In contrast, taxol was later shown to stabilise microtubular networks and was crucial for purifying microtubules and to characterise microtubule‐associated proteins.^[28]^ The microtubule‐stabilising effect of taxol impacts cell cycle progression by blocking chromosome segregation.^[29]^ Chemical inhibition of the cell cycle was subsequently exploited as an anticancer strategy.^[20b]^ This led to the successful clinical development of taxol (paclitaxel) and its synthetic derivative docetaxel as microtubule stabilising,^[30]^ and the *Vinca* alkaloids vinblastine and vincristine as microtubule destabilising agents.^[20b]^ Paclitaxel and docetaxel are approved to treat several solid tumours, including ovarian, breast and prostate cancer, among others.[^30^, ^31^] Vincristine and vinblastine are effective against several leukaemia types, Hodgkin's lymphoma and (small cell) lung cancer. Antimitotic agents remain an important pillar in cancer therapy to this day.^[32]^

Actin filaments may be similarly modulated as microtubules. Notably, cytochalasins and phalloidin exhibit destabilising and stabilising effects on actin filaments, respectively.^[26]^ Moreover, latrunculin A binds monomeric actin and accelerates actin depolymerisation.^[33]^ However, translating actin‐targeting agents failed due to adverse effects on cardiac and skeletal muscle tissue.^[20d]^ Small molecule modulators of IFs are generally less explored,^[34]^ as are cytoskeletal‐associated proteins, including plectin, myosins, and microtubule‐associated proteins (MAPs).

Here, we compile observations collected over the past decades that selected metal‐based drugs and candidate molecules are effective modulators of cytoskeletal proteins, as well as cytoskeleton‐associated proteins. We cover the metals platinum, gold, ruthenium, osmium, rhenium, iron, iridium, titanium, rhodium, palladium, copper, silver, zinc and bismuth. Cytoskeletal modulation may be achieved by indirect effects of the metal‐based candidate molecules or by direct interaction with a cytoskeleton(‐associated) target. First, the effects of these agents on microtubules, actin and IFs are separately discussed. Then, examples of validated cytoskeleton‐targeting will be specified. These studies underline that metal‐based candidate drugs by virtue of their covalent protein modification, charge or particular 3D ligand scaffold present a valuable solution to broadly modulate cytoskeleton (‐associated) proteins.

## 2 Effects of Metal‐based Candidate Drugs on Microtubules

Microtubules are the largest cytoskeletal system in living cells. They consist of heterodimeric α/β‐tubulins that bind GTP and impart a polarised filament structure.^[35]^ In general, microtubules can grow or collapse at both the plus and minus ends depending on the rate of GTP hydrolysis, but microtubular dynamics are faster at the plus end. Furthermore, cells possess microtubule‐organising centres made of γ‐tubulin that facilitate and accelerate microtubule assembly, including centrosomes near the nucleus or basal bodies near the cell membrane (Figure 1A).^[36]^ Interfering with microtubules during mitosis is a clinically used anticancer strategy to block cancer cell proliferation.[^20b^, ^35c^] There are six established drug binding sites on α/β‐tubulin corresponding to the cholchicine, taxane, vinca, maytansine, laulimalide and pironetin binding sites (Figure 1B and 1 C).^[20a]^ Interestingly, targeting β‐tubulin is more common compared to targeting α‐tubulin. The two microtubule stabilising sites of the taxanes and laulimalide are located on β‐tubulin, as are the destabilising sites corresponding to vinblastine and maytansine. The colchicine binding site is located at the interface of the α/β‐tubulin heterodimer and pironetin is fully located on α‐tubulin. These binding sites also lead to microtubule destabilisation.^[20a]^ A recent study has elaborated a total of 27 distinct potential binding pockets, including 11 unknown sites.^[37]^

Metal‐based candidate drugs were shown to be able to stabilise or destabilise microtubules, yet, insight into potential binding sites or molecular interactions is generally lacking. This complicates the establishment of structure‐activity relationships based on specific binding sites.

### 2.1 Stabilising Effects on Microtubules

**Figure 2:**
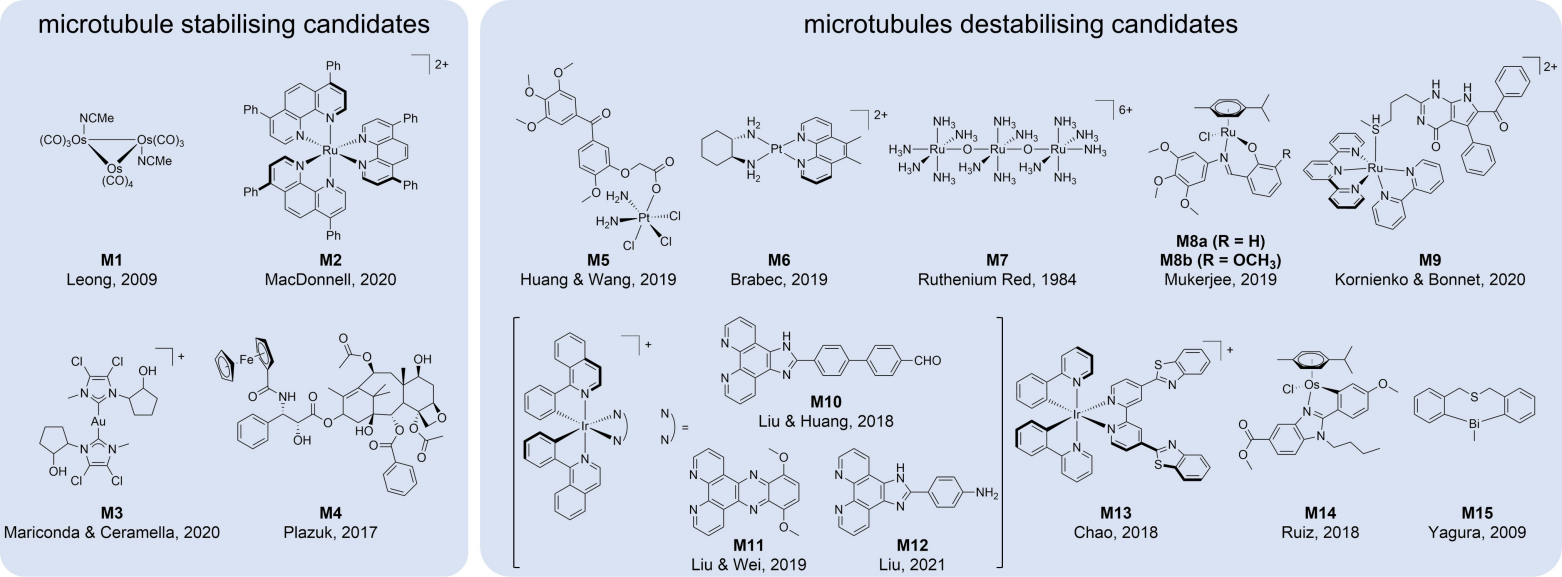
Chemical structures of metal‐based candidate drugs that stabilise **(M1**‐**M4**) or destabilise (**M5**‐**M15**) microtubules.

Only a few examples of metallodrug candidates are known to stabilise microtubules or promote their assembly. First, a trinuclear osmium carbonyl cluster with labile acetonitrile ligands (**M1**, Figure 2) caused a hyper‐stabilisation of microtubules, which subsequently led to mitotic catastrophe and apoptosis.^[38]^ This effect was hypothesised to be caused by the reactivity of **M1** against sulfhydryl groups, which was, however, not further investigated.

A ruthenium(II)‐based polypyridyl complex (**M2**) induced morphological changes and significantly altered microtubule networks in cancer cells.^[39]^ **M2** exhibited stimulating effects on tubulin polymerisation and ultimately led to microtubule network destruction and apoptosis.

A study involving gold(I) and silver(I) bis‐N‐heterocyclic (NHC) complexes in cancer cells identified **M3** as a promoter of tubulin polymerisation.^[40]^ Interestingly, a structurally similar gold(I) bis‐NHC complex (Figure 4, **A6**) displayed microtubule inhibiting properties. This opposing effect may be caused by differences in the ligand scaffold. The same authors demonstrated in a separate study that the two complexes **M3** and **A6** additionally inhibit actin polymerisation (s. below).^[41]^

Substituting individual phenyl rings of paclitaxel with ferrocene introduced redox activity to the microtubule stabiliser.^[42]^ Direct replacement of the phenyl with ferrocene resulted in the most cytotoxic derivatives, while bulkier substituents featured a >100‐fold lower cytotoxicity in some cases, which may be associated with a worse fit into the taxane binding pocket, as suggested by molecular docking studies. Furthermore, substitution of the A‐ring of paclitaxel (Figure 1C) yielded complex **M4** and retained the original cytotoxicity of paclitaxel, while substitution of the B‐ring resulted in reduced cytotoxic potency. Modulation of cytotoxicity by diastereomers was also observed. The direct replacement of the phenyl with ferrocene also resulted in a concentration‐dependent induction of tubulin polymerization.^[42]^

### 2.2 Destabilising Effects on Microtubules

Microtubule destabilising effects were evidenced more frequently for metal‐based candidate drugs, which may be explained by the higher number of potential destabilising binding sites on α/β‐tubulin.^[20a]^ Again, structural information about the binding interaction of microtubule‐destabilising metal‐based candidate molecules is currently lacking.

**Figure 3:**
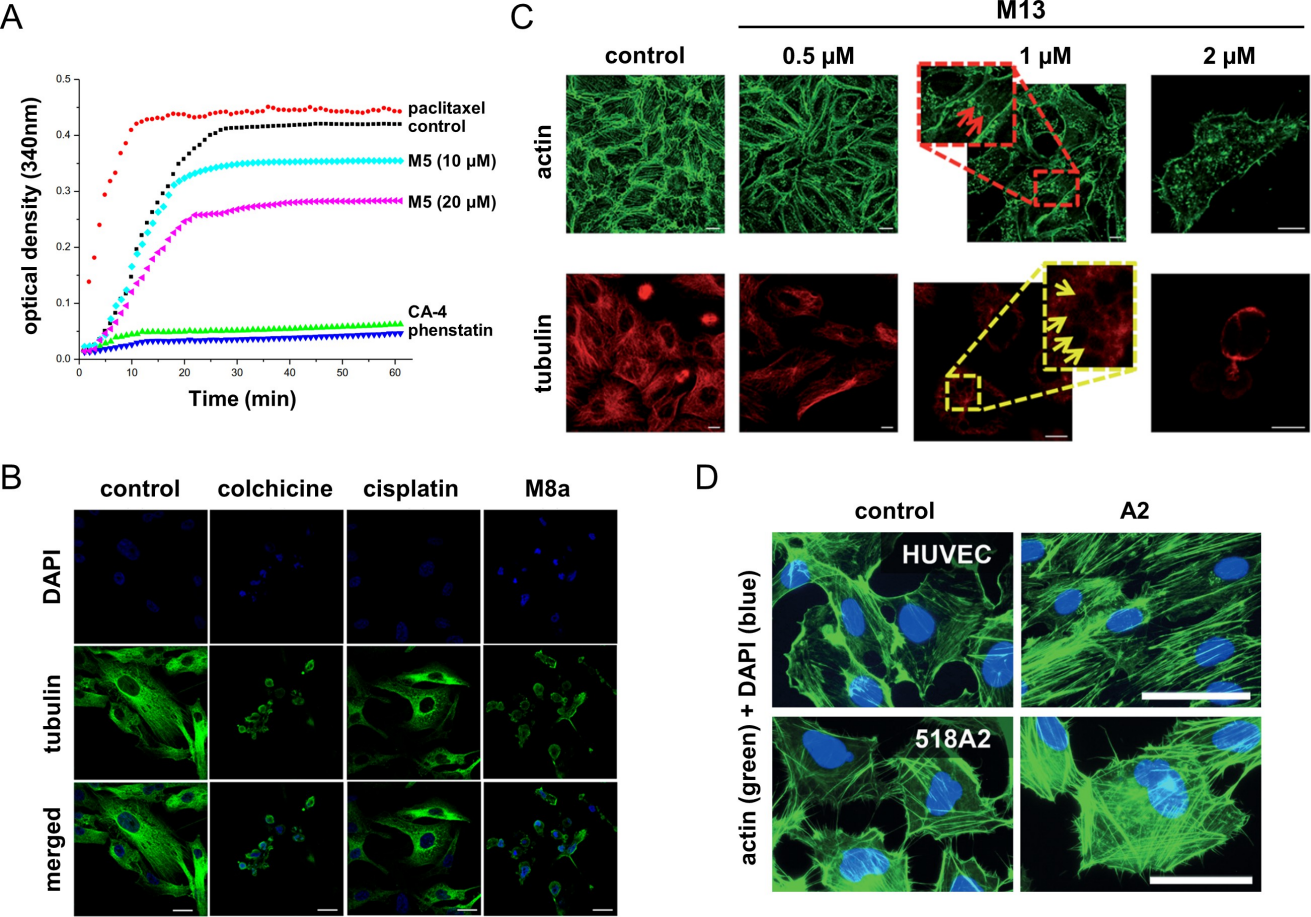
(**A**) Effect of the platinum(IV) phenstatin‐based **M5** on the polymerisation of tubulin *in situ* in comparison to positive controls for microtubule stabilisation (paclitaxel) and destabilisation (CA‐4 or phenstatin). **M5** inhibits tubulin polymerisation in a dose‐dependent manner, but is less potent than CA‐4 and phenstatin. Reproduced from ref. [43]. Copyright (2017), with permission from Elsevier. (**B**) Destabilising effect of the organoruthenium **M8a** on microtubules *in vitro*, visualised by immunofluorescence microscopy, including a positive (colchicine) and negative (cisplatin) control. **M8a** induces a similar collapse of microtubules as colchicine, while cisplatin does not affect the tubulin network. Reprinted with permission from ref. [47]. Copyright (2019) American Chemical Society. (**C**) The effect of the oncosis‐inducing iridium(III) derivative **M13** is shown on actin filaments and microtubules as evidenced by immunofluorescence microscopy. **M13** leads to a dose‐dependent collapse of both the actin filament and tubulin network. Reproduced from ref. [52]. Copyright (2018), with permission from the Royal Society of Chemistry. (**D**) The bimetallic **A2** leads to stabilisation of actin filaments as revealed by fluorescence microscopy. **A2** induced actin bundles that spanned the entire cells leading to a reorganisation of the actin network. This effect occurs in cancerous and healthy cells. Reproduced from ref. [66]. Copyright (2016), with permission from Wiley‐VCH.

Platinum(IV) complexes based on the cisplatin, oxaliplatin and DACH−Pt core, conjugated to phenstatin (**M5**), were investigated with respect to tubulin inhibition *in vitro* (Figure 3A).^[43]^ The newly synthesised analogues revealed decreased toxicity and increased cytotoxicity when compared to the parent compounds. Further experiments showed that phenstatin conjugated to the cisplatin core blocked tubulin polymerisation selectively, caused enhanced cell‐cycle arrest at the G2/M phase and subsequently induced apoptosis in NCI−H460 cells. Additionally, a NCI−H460 xenograft mouse model demonstrated a strongly reduced tumour size *in vivo*. In a follow‐up study, the same authors exchanged the phenstatin ligand for the colchicine‐mimic combretastatin A‐4 (CA‐4) and demonstrated again that conjugation to the cisplatin core exhibited a strong anticancer effect by microtubule disruption and significantly increased ROS levels in human cancer cell lines.^[44]^ In these studies, CA‐4 and phenstatin were also tested alone, as positive controls. Both are known to strongly inhibit tubulin polymerisation *via* binding to the colchicine binding site on β‐tubulin. In fact, their conjugation to platinum(IV) increased their cytotoxic potency in cancer cells while substantially decreasing the toxicity to normal cells.

Yet, CA‐4 and phenstatin were more potent destabilisers of microtubules compared to the platinum(IV) conjugates. Thus, the newly developed complex combines the tubulin inhibitory properties of the ligand with the DNA damaging effects of cisplatin, which is liberated upon intracellular reduction of the platinum(IV) prodrug. The substitution of the inert platinum(II) complex [platinum(1S,2S‐diaminocyclohexane)(5,6‐dimethyl‐1,10‐phenanthroline)]^2+^ (**M6**) decreased tubulin polymerisation and subsequently provoked its aggregation into nonfibrillar condensed structures.^[45]^ The authors additionally observed depolarised mitochondrial membrane potential (MMP), cell cycle arrest at the G2/M phase and apoptosis induction.

Already in 1984, the inhibitory effects of ruthenium red (**M7**) on microtubule assembly were characterised.^[46]^ Interestingly, it destabilised microtubules without evident structural or functional modifications. A study of ruthenium(II)‐cymene complexes containing an N,O‐bidentate trimethoxyphenylamine‐based Schiff base ligand in aggressive cancer cell lines revealed a dose‐dependent inhibition of tubulin polymerisation. The trimethoxyphenyl‐moiety was used as a fragment mimic of colchicine. The two tested derivatives, featuring either a hydrogen (**M8a**) or a methoxy‐group (**M8b**), were effective in depolymerising tubulins in MDA‐MB‐231 breast cancer cells (Figure 3B).^[47]^ Additionally, both complexes depolarised MMP with the induction of reactive oxygen species (ROS) at cytotoxic concentrations, and led to cell cycle arrest in the G2/M phase specific for tubulin inhibitors, followed by caspase‐cleavage and apoptosis. The strategy of photocaging was reported for a rigidin derivative, an experimental microtubule destabiliser (**M9**).^[48]^ To this end, a rigidin‐analogue containing a thioether moiety was coordinated to the ruthenium(II) photocage. Coordination to the ruthenium moiety inhibited the microtubule destabilising effect of the rigidin analogue, which, upon liberation by green light in water, is able to switch‐on microtubule destabilisation.

An organometallic iridium(III) complex (**M10**) was reported in 2018 that disrupted microtubules, amplified ROS and induced apoptosis when irradiated with white light.^[49]^ The 1‐phenylisoquinoline‐containing complex **M10** was slightly more cytotoxic in some cell lines compared to the more hydrophilic 2‐phenylpyridine analogue, but featured a comparable microtubule disrupting ability. A structurally similar iridium(III) complex also based on 1‐phenylisoquinoline (**M11**) was efficient in depolymerising microtubules leading to cell cycle arrest and, ultimately, apoptosis.^[50]^ Additionally, **M11** showed tumour inhibiting effects *in vivo*. A third organometallic iridium(III) complex based on 1‐phenylisoquinoline (**M12**) was described to destabilise microtubules and subsequently leading to cytoskeletal disruption, cell cycle arrest and induction of apoptosis.^[51]^ Furthermore, a cyclometalated 2‐phenylpyridine iridium(III) complex (**M13**) was shown to induce oncosis, which is a non‐apoptotic form of programmed cell death.^[52]^ This study revealed cytoskeletal breakdown and collapse of both microtubules and actin with increasing treatment concentrations (Figure 3C).

The osmium(II)‐arene complex containing a cyclometalated benzimidazole ligand (**M14**) blocked tubulin polymerisation, altered cell cycle progression and finally induced apoptosis. Additionally, this compound reduced intracellular ROS and NAD^+^ levels.^[53]^

Finally, a heterocyclic organobismuth(III) compound (**M15**) was reported to target tubulin and to induce cell cycle arrest in the G2/M phase and apoptosis in HeLa cells, similar to the above examples.^[54]^ The authors also observed increased expression levels of cyclin B and disassembly of microtubule networks in a dose‐dependent manner comparable to colchicine.

### 2.3 Other Effects on Microtubules

A proteomic investigation of ovarian cancer cells treated with a dithiocarbamate gold(III) complex revealed the down‐regulation of tubulin beta chain (TBB5).^[55]^ The authors described its involvement in chemoresistance and modulation of cell proliferation. A titanium(IV) salan complex was found to lead to a G2/M arrest. It targeted tubulin‐related signalling and induced alterations in the microtubular network.^[56]^ Interestingly, the compound provided partial protection against the cytotoxic effects of mitotic arrest‐inducing toxins that interact with tubulin like colcemid or docetaxel.

### 2.4 No Effects on Microtubules

Selected *C N N*‐cyclometalated NHC complexes were found to directly target vimentin, thus displaying antimetastatic and antimigratory properties *in vitro*. Interestingly, they did not affect microtubules.^[19a]^

Additionally, a cyclometalated iridium(III) phenantroline complex did not affect microtubular dynamics despite binding selectively to microtubules *in vitro*. This compound is used for live‐cell imaging of microtubule localisation.^[57]^ The authors demonstrated the efficacy of their photostable probe in multiple experimental levels and for multiple techniques.

### 2.5 Effects on Microtubule‐Binding Proteins

Microtubular dynamics are additionally regulated by microtubule‐associated proteins, *e. g*. stathmin and cofilin. Stathmin was identified as an oncoprotein, as a deficiency of it facilitates constant mitotic spindle formation. Stathmin binds to and destabilises tubulin protofilaments.^[58]^ Cofilin is not only associated with actin filaments (s. section 3.5.), but also regulates microtubules by competing with tau to inhibit tau‐induced microtubule assembly.^[59]^ Despite the important involvement of these regulating proteins in microtubule dynamics, the effect of metal‐based candidate drugs on these proteins has not yet been investigated.

   

In summary, microtubule (de‐)stabilisation by metal‐based candidate molecules is usually evaluated using a tubulin polymerisation assay in cell free medium, complemented by immunofluorescence microscopy of treated cells and the analysis of cell cycle arrest and apoptosis. This experimental framework provides a strong indication of whether the metal‐based candidate molecule interacts with microtubules. The current diversity of metals and ligand scaffolds that modulate microtubules suggest that multiple binding sites are indeed addressed. However, structural information about α/β‐tubulin binding remains to be elucidated. At least in the case of the observed examples of metal‐based microtubule stabilisers, the binding may occur in close proximity to the taxane or laulimalide binding sites.

## 3 Effects of Metal‐based Candidate Drugs on Actin

Actin filaments are a major component of the cytoskeleton and the contractile part of the muscle fibres.^[60]^ Globular G‐actin is synthesised as a single polypeptide chain with high‐affinity binding sites for Ca^2+^ ions and ATP. In the presence of K^+^ or Mg^2+^, the monomeric G‐actin polymerises spontaneously into actin filaments under ATP hydrolysis to form filamentous F‐actin. The process of polymerisation and depolymerisation within a cell is controlled by various actin‐binding proteins (ABP), *e. g*. filamin A or cofilin.^[61]^ Actin and the ABPs are involved in several phases of carcinogenesis. They may support apoptosis inhibition or facilitate tumour invasion.^[62]^

Actin features at least three established drug binding sites that either lead to stabilisation or destabilisation of actin filaments (Figure 1D). Phalloidin stabilises filamentous actin, where it binds specifically at the interface between two actin monomers (Figure 1E).^[63]^ The latrunculin and cytochalasin binding sites in close proximity to the ATP binding site lead to destabilisation of actin. Latrunculin A associates with actin monomers and prevents their polymerisation into filaments (Figure 1E).^[64]^ In contrast, cytochalasin D binds to the barbed end of actin filaments and interferes with actin dynamics leading to their depolymerisation.^[65]^

In the 1970s, OsO_4_ was employed for the fixation of tissue sections prior to electron microscopy. Beyond the mere fixation, it was realised that the abundantly observed cross‐linked actin microfilaments did not represent the actual state of the sample, but were an artefact of the interaction between OsO_4_ and actin during sample processing.^[67]^ Since then, numerous investigations reported stabilising and destabilising effects of metal‐based candidate drugs on actin. Early studies evaluated the interaction of cisplatin^[68]^ and DACH‐derivatives^[22]^ with isolated G‐actin. However, such studies on isolated proteins tend to overestimate the specificity of the metal‐protein interaction. This was also indicated by numerous mass spectrometry‐based interaction studies.^[69]^ Competitive experiments provide a more realistic estimation of binding selectivity.^[70]^

### 3.1 Stabilising Effects on Actin

**Figure 4:**
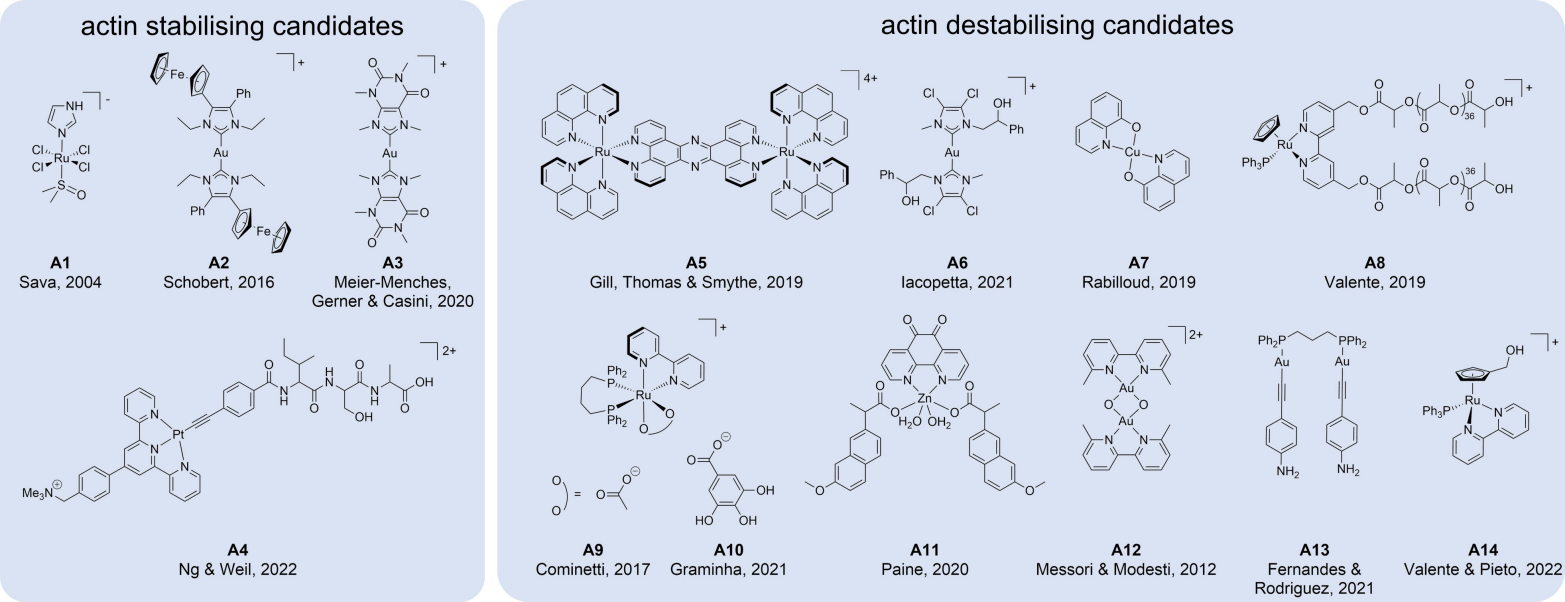
Chemical structures of metal‐based candidate drugs that stabilise **(A1**‐**A4**) or destabilise (**A5**‐**A14**) actin filaments.

Metal‐based candidate drugs with stabilising effects on actin were only scarcely reported. Sava *et al*. reported that low dose treatment with complex NAMI−A (**A1**, Figure 4) resulted in pro‐adhesive effects in two human carcinoma cells.^[71]^ NAMI−A was found to rapidly react with one or more membrane‐located targets thus initiating the nucleation of actin in the cytoplasm and its elongation to microfilaments, causing increased adhesion in tumour cells. The treatment led to strong, irreversible cross‐linking with the substrate and finally immobilisation. This inhibited cell migration and was suggested to be responsible for the antimetastatic activity of this clinically evaluated compound.^[71]^

Actin‐stabilising effects were also evidenced for a heterobimetallic ferrocenyl‐conjugated gold(I) NHC complex (**A2**), where the ligand system was actually intended to mimic the microtubule targeting CA‐4.^[66]^ **A2** and the corresponding mono‐carbene derivative were studied for their antivascular and antiproliferative activity. The complexes were found to cause filamentous F‐actin to form bundles spanning the cells, thus reorganising the actin cytoskeleton in non‐malignant HUVEC as well as in 518 A2 melanoma cells, although the effect was more prominent in HUVEC cells (Figure 3D). The authors described, that the reorganisation of F‐actin is accompanied by a reduced cell migration upon treatment and a cell cycle arrest in the G0/G1 phase.^[66]^

A gold(I) bis‐NHC complex with 9‐methylcaffeine‐8‐ylidene ligands (**A3**) was shown to induce a Nrf2‐stress response in ovarian cancer cells. This was associated with increased ROS and the formation of actin stress fibres in the cell periphery.^[72]^

A recent study showed that an intracellularly released linear platinum(II)‐tripeptide (**A4**) forms nanofibers, which was accompanied by inhibition of cell metabolic processes such as aerobic glycolysis and oxidative phosphorylation and thus, ATP production. Deregulated ATP production seemed to lead to rearrangement of actin along the cell membrane.^[73]^

### 3.2 Destabilising Effects on Actin

Destabilising effects of metal‐based candidate molecules on actin were more frequently observed and range from inhibition of polymerisation,^[19b]^ to disruption[^52^, ^74^] and even degradation.^[75]^

F‐actin remodelling was associated with platinum resistance. While cisplatin‐treatment increased the stiffness of sensitive ovarian cancer cells in a dose‐dependent manner, no such effect was observed for the resistant ovarian cancer cells.^[76]^ The increase in stiffness was associated with disruption of actin polymerisation.

The binuclear polypyridyl ruthenium(II) complex (RuRuPhen, **A5**) was demonstrated to inhibit F‐actin polymerisation without depolymerising pre‐existing actin filaments (s. below).^[19b]^ Similarly, the two Au(I) bis‐NHC complexes (**M3**, **A6**) mentioned above with opposing effects on microtubules, were both found to inhibit actin polymerisation without affecting filament depolymerisation.^[41]^

Alterations on the actin cytoskeleton were evidenced for a number of metallodrugs such as copper(II)‐hydroxyquinoline complex (**A7**),^[77]^ cyclopentadienyl ruthenium(II) conjugated to bipyridine polylactide chains (**A8**),^[78]^ and inert ruthenium(II) complexes (**A9, A10**),^[79]^ as well as zinc(II)–NSAID complexes with phendione and naproxen (**A11**).^[80]^ The different research groups commonly described that the treatment did not only result in a change in the cellular morphology and reduction of cell size, but crucially, in a loss of actin density. Of note, Dalzon *et al*. showed that the copper complex **A7** was necessary to alter the actin cytoskeleton, as the ligand alone was inactive.^[77]^ In contrast, the activity of the zinc(II)‐NSAID complexes with phendione and naproxen was mainly mediated by phendione as the other components did not reveal any substantial effect alone.^[80]^ The dinuclear dioxygen‐bridged gold(III) complex Auoxo6 (**A12**),^[81]^ as well as a linear dinuclear gold(I) complex containing a 4‐ethynylaniline ligand and a bridging diphosphine (**A13**),^[82]^ were described as modulators of actin aggregation including a reduced expression of actin isoforms after cell treatment.

An actin depolymerising effect of metal‐based candidate molecules was observed in some studies, including ruthenium(II) cyclopentadienyl complexes with bipyridine ligands (**A14**)^[83]^ and a gold(I) thiazolylalanine bis‐NHC complex.^[74]^ Actin depolymerisation was generally followed by apoptosis‐induction. In fact, these actin‐disruptive effects were similar to those of the non‐metallic anti‐actin drug candidate TR100,^[84]^ which was shown to target tropomyosin, an integral part of the actin filament. Interestingly, this molecule showed no effects on cardiac actin filaments, highlighting that tropomyosin‐targeting may be useful to circumvent actin‐related cardiac toxicity.^[84]^ Moreover, it seems that concomitant actin and microtubule collapse may lead to oncosis as evidenced for the iridium(III) complex **M13**.^[52]^

### 3.3 Other Effects on Actin

Additional metal‐based candidate drugs were identified that affected actin, but it was uncertain whether they act via stabilising or destabilising effects. On the one hand, iridium(III) complexes containing benzimidazole‐ligands were found to co‐localise with the actin cortex.^[85]^ On the other hand, the interaction of decavanadate (V_10_) and the more stable, isostructural decaniobate (Nb_10_) with G‐actin were evaluated by *in silico* approaches based on experimental data.^[86]^ It was revealed that both substances lead to a structural reorganisation of G‐actin but cellular down‐stream effects were not evaluated in this report.

### 3.4 No Effect on Actin

It is worth mentioning that there are also reports of metal‐based candidate molecules that did not show any impact on the actin cytoskeleton. Among those were rhenium(I) tricarbonyl phenanthroline complexes,^[87]^ gold porphyrin compounds tethered to organotin(IV) entities^[88]^ and a mercury(I) thiazolylalanine bis‐NHC complex whose isostructural gold analogue actually destabilises actin.^[74]^

### 3.5 Effects on Actin‐Binding Proteins

*Filamin*. The organisation of the actin cytoskeleton is further supported by ABPs, including filamins. These are large proteins that provide a backbone for >90 proteins of signalling pathways like GTPases, phosphatases, tyrosine kinases as well as integrins, and other adhesive receptors. By doing so, the filamins associate the signalling pathways to the adhesive receptors and to the cytoskeletal actin, serving as a cross‐linker.[^61b^, ^62^] Several proteomic investigations highlighted reduced expression of filamin A upon treatment with carboplatin,^[89]^ the half‐sandwich ruthenium(II)‐arene compound RAPTA−T,^[90]^ or a gold(I) bis‐NHC complex with caffeine ligands.^[72]^

Additionally, it was found that cancer cells were more sensitive to cisplatin when filamin A expression was reduced.^[91]^ This slowed down the repair of single and double strand breaks, inter‐strand cross‐links and enhanced DNA damage after treatment.

*Cofilin*. Cofilin is a further actin‐controlling protein.^[92]^ When cells are under oxidative stress, cysteine residues on cofilin are oxidised, resulting in the formation of cofilin dimers that lead to cross‐linking of actin filaments.^[93]^ These stable actin filaments save cellular ATP because it is not needed for polymerisation, which in turn contributes to the faster recovery of the cell from oxidative stress. As such, cofilin might play an important role in redox active metal‐based candidate molecules.

A differential regulation of cofilin‐1 was observed in cisplatin‐sensitive and ‐resistant A2780 ovarian cancer cells lines, with an increased expression level up to 20‐fold in the cisplatin‐resistant compared to the parent cell line. The authors attributed the platinum resistance to the actin‐^[94]^ and apoptosis‐regulating^[95]^ properties of cofilin.

   

In summary, metal‐based candidate drugs were shown to stabilise or destabilise actin, suggesting that these agents target several binding sites on either monomeric or filamentous actin. These metal‐based agents are often electrophilic and metallate intracellular nucleophiles. This may be sensed by different cellular stress response systems, *e. g*. cofilin to save ATP equivalence or the Nrf2 stress response to increased glutathione concentrations. Due to the intricate connection between Nrf2‐detoxification system,^[96]^ cofilin, and the actin cytoskeleton, it is often challenging to separate the actual metal‐mediated effect on actin from actin stress responses. Such indirect cellular responses need to be accounted for in future experimental designs. Similar to the situation with microtubules, insight into the direct interaction at the molecular level is lacking, but crucial to advance the rational design of metal‐based modulators of the cytoskeleton and its proteins.

## 4 Effects of Metal‐based Candidate Drugs on Intermediate Filaments

Several processes that provide for cell and tissue integrity or cellular stress responses and adaptations are controlled by IFs. These filaments are grouped into 5 classes according to their structure and function: type I IFs include acidic cytokeratins (CK) CK9‐CK20, type II comprises basic cytokeratins CK1‐CK8, type III encompasses vimentin, desmin and peripherin, type IV consists of neurofilaments, nestin and internexin, type V IFs sum‐up the lamins. IFs support cellular architecture and function, for example, intracellular structures or peripheral cell junctions.^[97]^ In the following subsections, we focus on drug effects on keratins, vimentin as well as on the cytolinker plectin.^[98]^ It is noteworthy that complete molecular structures of either of these proteins remain to be solved thus complicating drug discovery efforts.

### 4.1 Effects on Keratins

In human epithelial cells, pairs of type I and II cytokeratins form IFs that constitute the cellular scaffold against mechanical stress and thus, control tissue integrity. They are encoded by 54 genes, whereby cytokeratin mutations may result in severe diseases, *e. g*. epidermolysis bullosa simplex, a blistering skin disease.[^97b^, ^99^] In principle, the diversity and tissue‐specificity of keratins make them attractive drug targets with potentially selective effects and acceptable safety profiles.^[20e]^

In the 1960s, OsO_4_ was not only used as a contrast agent for microtubules, but also for keratins in nails or hair/wool. In contrast to the observation of sample processing artefacts during microtubule visualisation, pre‐treatment with thioglycolate allowed the correct detection of filament matrix complexes of α‐keratin in human hair. Even prolonged pre‐treatment had almost no destructive effect on hair samples, although it may be noted that the study authors tested highly rigid, cell‐free samples.^[100]^ The dinuclear gold(III) compound **A12** was found to reduce the expression of cytoskeletal proteins in cisplatin‐resistant ovarian cancer cells (A2780/R), especially two keratins (type I and II), but this effect was not further evaluated.^[81]^ Moreover, cytokeratin 18 (CK18) was significantly upregulated upon treatment of HCT116 colon cancer cells with the organoruthenium complex plecstatin‐1 and affected the IF network.^[4c]^

### 4.2 Effects on Vimentin

Vimentin is a type III IF protein expressed in almost every human tissue. It exhibits similar functions as keratins,^[97a]^ but additionally, is a key player in the EMT.^[101]^ The EMT describes a change in cancer phenotype from an immobile towards a more motile cell state that enables metastasis.^[102]^ This process is associated with an enhanced expression of vimentin at the cost of E‐cadherins. Due to the identification of EMT as a key process in cancer pathophysiology, the last decade saw an increased interest in modulating vimentin. Metal‐free examples include the steroidal lactone withaferin A, which is known to directly interact with vimentin, promoting its degradation and subsequently inducing apoptosis,^[103]^ and FiVe1, which leads to mitotic disruption in mesenchymal cancer cells.^[104]^ To the best of our knowledge, experimental molecular structures have not yet been reported for these two modulators interacting with vimentin.

Möltgen *et al*. investigated binding partners of cisplatin in ovarian and oxaliplatin in colorectal cancer cells including their respective platinum‐resistant homologous cells. For this purpose, they used a fluorescent cisplatin analogue containing BODIPY.^[105]^ Interestingly, the authors determined that the compound co‐localised with vimentin in ovarian cancer cells. Inhibition of vimentin by the organic FiVe1 significantly sensitised the ovarian cancer cells towards cisplatin and led to higher rates of apoptosis even in the resistant cancer cells.

**Figure 5:**
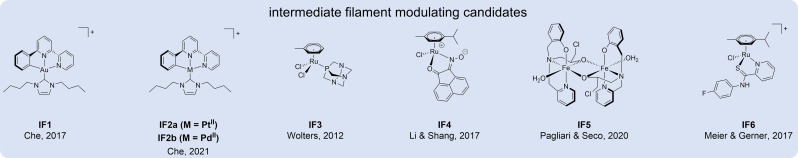
Chemical structures of metal‐based candidate drugs that modulate intermediate filaments (**IF1**‐**IF5**) and the intermediate‐filament binding protein plectin (**IF6**).

In 2017, CM Che reported a pincer cyclometalated gold(III) NHC complex (**IF1**, Figure 5) that led to vimentin filament degradation in a dose‐dependent manner, and this was associated with enhanced activation of apoptosis‐inducing proteins, such as CASP3 and CASP9.^[106]^ The authors also observed similar results with platinum(II) and palladium(II) analogues (**IF2a** and **IF2b**) revealing a metal‐independent effect of the complexes on vimentin. In a follow‐up study they identified **IF2a** to potently destroy vimentin networks and to decrease vimentin expression. Additionally, the compound exhibited antitumour activity *in vivo* and inhibited tumour metastasis.^[19a]^

The cytotoxicity of the above mentioned Au(I) NHC complex (**A6**) was found to be associated with targeting topoisomerases,^[107]^ but also with a downregulation of vimentin.

*In vitro* investigations on the anticancer potential of ruthenium(arene) complexes, namely the antimetastatic RAPTA−T (**IF3**)^[108]^ and derivatives containing α‐dicarbonylmonoxime ligands (**IF4**),^[109]^ witnessed a down‐regulation of vimentin protein expression in both cases. The authors of the latter study found a concomitant increase in E‐cadherin protein expression, which would explain its antimigratory activity.^[109]^ In contrast, the organoruthenium plecstatin‐1 (**IF6**) did not affect E‐cadherin, nor vimentin on the protein level in tumour spheroids as revealed by fluorescence microscopy.^[110]^

Treating H4 glioma cells with tetradentate iron(III) complexes (**IF5**) resulted in non‐significantly reduced vimentin mRNA expression accompanied by non‐significantly increased expression of E‐cadherin mRNA‐levels.^[111]^ The increase in E‐cadherin was significant for the analogous copper complex. In addition, the authors observed a significant reduction in ROS levels as **IF5** mimics cellular metalloenzymes such as superoxide dismutase, superoxide oxidase and catalase, thus exhibiting antioxidant behaviour. Yet, the reduced vimentin mRNA expression upon treatment was accompanied by a reduced invasiveness in 3D spheroids.^[111]^

A similar downregulation of vimentin expression level was also found in fluorescence microscopy for the mentioned zinc(II)‐NSAID complex (**A11**)^[80]^

### 4.3 Effects on IF‐Binding Proteins

*Plectin*. Plectin is a scaffold protein and cytolinker that anchors IFs to specific sites to support cellular stress resilience, function and the organisation of peripheral cell junctions.^[98]^ Plectin not only anchors IFs, but acts as a scaffold to connect other cytoskeletal proteins such as actin or tubulin.^[97a]^

Although the organoruthenium plecstatin‐1 (**IF6**) was identified as a plectin targeting metal‐based candidate, no significant effects on the distribution or expression of plectin were observed upon treatment.[^18b^, ^23^] Fitting to its role as a cytolinker, plectin‐targeting by plecstatin‐1 was shown to reduce the invasiveness of cancer cells in a 3D *in vitro* model.^[18b]^

   

In summary, modulating *hard‐to‐drug* IF or IF‐binding proteins that are devoid of cofactor or other binding sites by metal‐based candidate molecules emerged as a promising anticancer strategy. IF and IF‐binding proteins are crucially involved in EMT and tumour invasion. With **IF2a** and **IF6**, there are currently two metal‐based candidate molecules that directly target vimentin and plectin, respectively. Further experimental information about their molecular interaction and comprehensive analysis of their down‐stream effects will enable a rational design of agents to modulate the biological function of these proteins.

## 5 Validated Cytoskeleton‐Targeting Metal‐based Candidate Drugs

Despite the considerable number of studies focussing on metal‐based candidate drugs impacting the cytoskeleton, there are currently only a few metal compounds with identified *and* validated cytoskeletal or scaffold protein targets including actin, vimentin and plectin. With the exception of some microtubule‐targeted agents or mimics conjugated to metal‐moieties, it is often not conclusively evaluated whether the observed effects stem from direct interaction of the metal with cytoskeletal proteins or from indirect cellular responses. We focus on those cytoskeletal targets, that have been validated as direct interactors of metal‐based candidate drugs in experiments that considered the cellular complexity. For example, the identification of vimentin and plectin, as direct targets, was enabled by mass spectrometry‐based methods. For details about this technique, the interested reader is referred to other reviews.[^5^, ^17a^, ^17c^]

### 5.1 Actin

**Figure 6:**
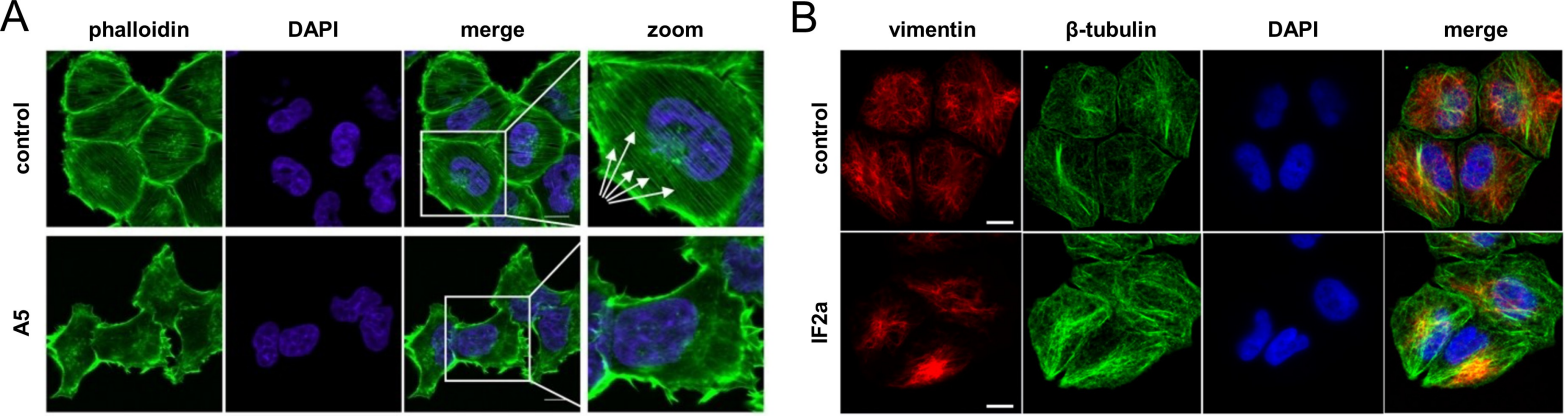
(**A**) The dinuclear bipyridyl ruthenium complex (RuRuPhen, **A5**) targets the actin cytoskeleton. **A5** inhibits actin polymerisation without depolymerising pre‐existing actin filaments leading to the degradation of cytoplasmic actin. Reproduced from ref. [19b]. Copyright (2022), with permission from Wiley‐VCH under a Creative Commons CC BY 4.0 license. (**B**) The cyclometalated platinum(II) derivative **IF2a** disturbs the intermediate filament by directly targeting vimentin, thus destroying IF networks, but does not affect the tubulin network considerably. **IF2a** also exhibits anti‐metastatic effects *in vivo*. Reproduced from ref. [19a] with permission from PNAS.

The dinuclear bipyridyl ruthenium complex (RuRuPhen, **A5**) was recently found to inhibit the formation of F‐actin without disassembling the already existing actin filaments (Figure 6A).^[19b]^ Polymerisation of pyrene‐labelled G‐actin was examined in a cell‐free setting, showing a concentration‐dependent decrease in the polymerisation rate. Next, by employing docking studies to investigate the binding sites of **A5** on G‐actin revealed that the compound is too large for any known binding pocket, but instead may affect filament formation by binding to protein interfaces. Live‐cell uptake experiments showed that the dinuclear complex preferably co‐localised with actin filaments over microtubules. Importantly, stabilisation of midbodies during cytokinesis was observed with time lapse videos and indirect immunofluorescence. However, since this did not lead to binuclear cells, interference with cytokinesis in its late stage by the specific modulation of actin was suggested as a MoA of this compound. In fact, the group uncovered a dual MoA of **A5**, which inhibits cytokinesis in a late phase and perturbs the cytoskeletal integrity of transfected cells.

### 5.2 Vimentin

The pincer gold(III) NHC complex (**IF1**) was identified to target multiple proteins, including vimentin.^[106]^ By employing an integrated proteomic LC–MS approach it was shown that **IF1**, as well as its Pt analogue (**IF2a**), had similar engagement patterns in the investigated cell lines. Subsequently, a biotin‐streptavidin pulldown coupled with western blotting validated vimentin and five other proteins as targets for **IF1**. In the follow‐up study, the Pt‐derivative **IF2a** was further examined and its interaction with vimentin was confirmed (Figure 6B).^[19a]^ Among others, the direct interaction of **IF2a** with the rod domain of vimentin was evaluated by NMR, surface plasmon resonance and molecular dynamic simulations. It was revealed that the association of **IF2a** leads to a bending of the vimentin rod domain in the region of Y276‐W290. Furthermore, the authors showed the importance of the ligand scaffold in directing the non‐covalent binding to vimentin. *In vitro* and *in vivo* assays, as well as spectroscopic methods were applied to investigate the compounds’ antimetastatic properties. **IF2a** was better tolerated *in vivo* and showed an enhanced renal clearance compared to cisplatin.

### 5.3 Plectin

The *S,N*‐bidentate pyridinecarbothioamide ruthenium(arene) complex (plecstatin‐1, **IF6**) was found to directly target the scaffold protein plectin and thus, represents a first‐in‐class modulator. The target selectivity was thoroughly investigated in integrated proteomics‐based target‐profiling approaches.[^4c^, ^18b^] First, pulldown experiments identified plectin as the main target of **IF6**, which was validated by immunofluorescence microscopy in plectin wildtype and knock‐out keratinocytes.^[18b]^ These experiments revealed, that after treatment of wildtype cells with **IF6**, the microtubules reorganised into dense circular networks wrapping around the nucleus and arresting the cells in the G0/G1 phase of cell cycle. This effect was entirely absent in the knock‐out cells. Interestingly, plecstatin‐1 treatment was accompanied by a reduction of acetylated microtubules in both wildtype and knock‐out cells supporting the fact that microtubules became more dynamic upon plecstatin‐1 treatment. Plecstatin‐1 is a metallo‐prodrug that requires activation by hydrolysis of the metal‐chlorido bond. Target profiling experiments with the metallo‐prodrug and the activated hydrolysed form are capable of covalently modifying histidines and revealed that this activation step is necessary for an efficient target interaction with plectin.^[4c]^ Additionally, a plecstatin‐1 analogue with a hydrogen bond donor (−OH) instead of a hydrogen bond acceptor (‐F) was synthesised and resulted in a reduced plectin interaction.^[4c]^ Prechova *et al*. used plecstatin‐1 to investigate plectin‐mediated cytosekeletal crosstalk and its effect on cell tension as well as cohesion. Therein, plecstatin‐1 was unequivocally shown to induce a phenotype that strongly resembled the one of genetic plectin knock‐out.^[23]^ Likewise, upon mechanical stretching, the knock‐out and plecstatin‐1‐treated wildtype cells showed significantly more fragments compared to untreated wildtype cells indicating that plecstatin‐1 treatment also leads to a loss‐of‐function that manifests at the level of the IF network. Thus, Plectin targeting might be utilised, as a potential treatment option, to inhibit cancer motility and invasiveness.

## 6 Summary and Outlook

The cytoskeleton represents an important drug target, which is clinically exploited in the treatment of cancer since many years. An increased interest in MoA deconvolution in the field of metal‐based anticancer agents revealed numerous candidate drugs that modulate the cytoskeleton. In fact, direct morphological and indirect molecular experiments underlined the potential to modify the three main cytoskeletal networks, including microtubules, actin filaments and IFs, as well as cytoskeletal binding proteins, *e. g*. plectin. Metal‐based candidate drugs promise to be valuable for targeting even hard‐to‐drug targets, such as intermediate filaments, as they can covalently modify targets. Moreover, their ligand scaffold forms particular three‐dimensional structures that are not easily available by conventional organic scaffolds.

Metal‐based candidate molecules from various metal families featuring diverse ligand scaffolds were shown to affect the cytoskeletal network or the expression of its building blocks. Yet, no clear‐cut structure‐activity relationships emerged so far, mainly due to a lack of information about the exact binding sites and the molecular interactions. Interestingly, these agents differentially modulate the cytoskeleton and are able to either stabilise or destabilise actin filaments and microtubules. Where comparison was possible, the ligand scaffold determined this effect more strongly compared to the metal centre.

Specific metal‐based candidate drugs based on ruthenium(II) polypyridine, cyclometalated platinum(II) NHC and ruthenium(II) arene were shown to directly engage with cytoskeletal and cytoskeleton‐associated proteins, including actin, vimentin and plectin, respectively. This suggests that metal‐based candidates may be particularly suited to modulate cytoskeletal proteins. However, it may be noted that early compound screenings for microtubule inhibitors were known to produce relatively high hit rates probably because of the many potential binding sites on tubulin.^[112]^ Similarly, simple polymerisation assays or experiments with purified proteins may tend to overestimate the specificity of interaction compared to the cellular complexity. The discovery of metal‐based interactors of cytoskeletal proteins should therefore necessarily be followed by competitive assays and in‐depth MoA validation. Alternatively, a systematic analysis of multiple families of metal‐based candidate drugs by high‐content image‐based assays might be worthwhile to determine predictors of cytoskeletal engagement, *e. g*. by the Cell Painting assay.^[113]^ Also, detailed molecular information about the binding sites and molecular interactions of metal‐based candidate drugs with cytoskeletal and cytoskeleton‐associated proteins either from experiments or molecular dynamic simulations will be crucial to establish site‐specific structure‐activity relationships in the future. Additionally, elucidating the modulation of post‐translational modifications of cytoskeletal and cytoskeleton‐associated) proteins will help in categorising the effect of metal‐based candidate drugs on cytoskeletal dynamics.

Finally, immune modulation by metal‐based candidate drugs is an emerging field and several agents were shown to induce immunogenic cell death.^[114]^ As the involvement of the cytoskeleton as a functional platform in immune responses is being uncovered, it might be worthwhile investigating the potential of cytoskeletal modulation by these candidate drugs for immunotherapy.

## Conflict of interest

The authors declare no conflict of interest.

## Biographical Information

*Yasmin Borutzki trained as a chemical‐technical assistant and then studied applied chemistry (BSc) and toxicology (MSc), completing each with a thesis on method development in targeted metabolomics. She is currently researching the post‐genomic modes of action of metal‐based (cancer) drug candidates. Other interests include cell culture, bioanalytical assays in combination with targeted and untargeted omics techniques for a global investigation of mechanisms of pathophysiological states or metal‐based drug candidates*.

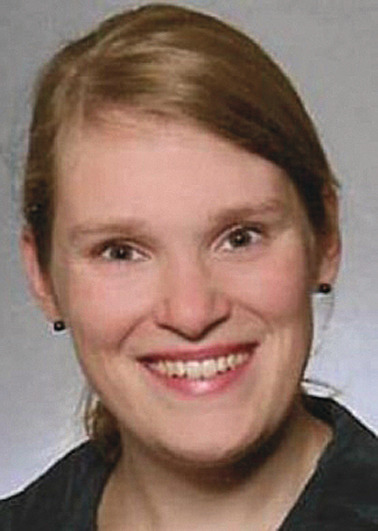

## Biographical Information

*Lukas Skos obtained his master's degree in Chemistry in 2021 at the University of Vienna in Austria and subsequently joined the research groups of Prof. Gerner and Prof. Meier‐Menches as a PhD student. His work focuses on the establishment of an affinity enrichment method for the selenoproteome via immobilised gold complexes for proteomics. Further interests are cell culture and the investigation of pathophysiological mechanisms as well as metal‐based drug candidates*.

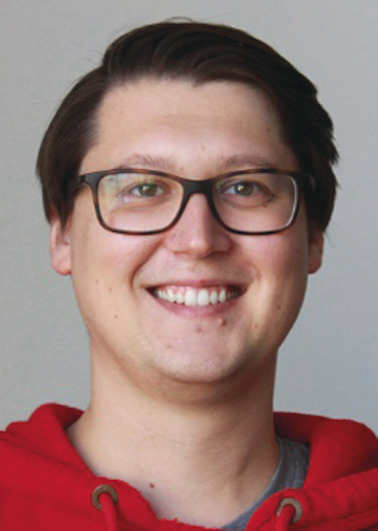

## Biographical Information

*Christopher Gerner heads the Institute of Analytical Chemistry (Faculty of Chemistry, University of Vienna) and the Joint Metabolome Facility. After several years in cancer research, the biochemist expanded his research field into proteomics. Since 2012, he has been leading the Bioanalytics group as a full professor with a main focus on mass spectrometry‐based post‐genomic research. His research includes multilevel omics studies and he employs these strategies to characterise disease pathophysiology*.

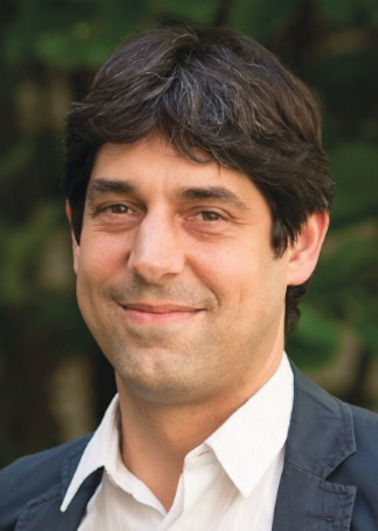

## Biographical Information

*Samuel Meier‐Menches holds a tenure‐track position at the Faculty of Chemistry of the University of Vienna and the Joint Metabolome Facility since 2021. He conducts research in an interdisciplinary environment on drug effects from preclinical models to patients based on proteomic and metabolomic analyses. One of his research interests centres on the generation of novel chemical probes to map the target landscapes of (metal‐based) candidate drugs*.